# Patterns of Street Food Purchase in Cities From Central Asia

**DOI:** 10.3389/fnut.2022.925771

**Published:** 2022-06-24

**Authors:** Sofia Sousa, Inês Lança de Morais, Gabriela Albuquerque, Marcello Gelormini, Susana Casal, Olívia Pinho, Carla Motta, Albertino Damasceno, Pedro Moreira, João Breda, Nuno Lunet, Patrícia Padrão

**Affiliations:** ^1^EPIUnit - Instituto de Saúde Pública, Universidade do Porto, Porto, Portugal; ^2^Laboratório para a Investigação Integrativa e Translacional em Saúde Populacional (ITR), Porto, Portugal; ^3^Faculdade de Ciências da Nutrição e Alimentação da Universidade do Porto, Porto, Portugal; ^4^Nutrition, Physical Activity and Obesity Programme, Division of Noncommunicable Diseases and Life-Course, World Health Organization (WHO) Regional Office for Europe, Copenhagen, Denmark; ^5^LAQV/REQUIMTE, Laboratório de Bromatologia e Hidrologia, Faculdade de Farmácia, Universidade do Porto, Porto, Portugal; ^6^Departamento de Alimentação e Nutrição, Instituto Nacional de Saúde Doutor Ricardo Jorge (INSA), Lisboa, Portugal; ^7^Departamento de Ciências da Saúde Pública e Forenses e Educação Médica, Faculdade de Medicina da Universidade do Porto, Porto, Portugal; ^8^Faculdade de Medicina, Universidade Eduardo Mondlane, Maputo, Mozambique; ^9^Centro de Investigação em Atividade Física, Saúde e Lazer, Universidade do Porto, Porto, Portugal; ^10^WHO Regional Office for Europe, Athens, Greece

**Keywords:** street food, ready-to-eat food, purchasing patterns, food choice, nutritional value, Central Asia, low- and middle-income countries, nutrition transition

## Abstract

Street food makes a significant contribution to the diet of many dwellers in low- and middle-income countries and its trade is a well-developed activity in the central Asian region. However, data on its purchase and nutritional value is still scarce. This study aimed to describe street food purchasing patterns in central Asia, according to time and place of purchase. A multicentre cross-sectional study was conducted in 2016/2017 in the main urban areas of four central Asian countries: *Dushanbe* (Tajikistan), *Bishkek* (Kyrgyzstan), *Ashgabat* (Turkmenistan) and *Almaty* (Kazakhstan). Street food markets (*n* = 34) and vending sites (*n* = 390) were selected by random and systematic sampling procedures. Data on the purchased foods and beverages were collected by direct observation. Time and geographic location of the purchases was registered, and their nutritional composition was estimated. A total of 714 customers, who bought 852 foods, were observed. Customers' influx, buying rate and purchase of industrial food were higher in city centers compared to the outskirts (median: 4.0 vs. 2.0 customers/10 min, *p* < 0.001; 5.0 vs. 2.0 food items/10 min, *p* < 0.001; 36.2 vs. 28.7%, *p* = 0.004). Tea, coffee, bread and savory pastries were most frequently purchased in the early morning, bread, main dishes and savory pastries during lunchtime, and industrial products in the mid-morning and mid-afternoon periods. Energy and macronutrient density was highest at 11:00–12:00 and lowest at 09:00–10:00. Purchases were smaller but more energy-dense in city centers, and higher in saturated and *trans*-fat in the peripheries. This work provides an overview of the street food buying habits in these cities, which in turn reflect local food culture. These findings from the main urban areas of four low- and middle-income countries which are currently under nutrition transition can be useful when designing public health interventions customized to the specificities of these food environments and their customers.

## Introduction

In the last few decades, most low- and middle-income countries (LMIC) have been experiencing rapid socioeconomic development, accompanied by a wide and abrupt increase in urban agglomerations and industrialization (1, 2). In central Asia, countries have undergone remarkable gross domestic product growth during the last decade, averaging around 5% per year (3), and urban population has grown from ~25 million to more than 35.5 million people between 2000 and 2019, the latter representing almost half of the total central Asian population (4).

These major demographic and economic changes have led to marked lifestyle shifts, such as the growing participation of women in the workforce to the detriment of housework, and the increase in time spent working or commuting. This has hindered household food preparation and consumption, making away-from-home food sources, such as street-vended foods, to be seen by the urban dwellers as excellent alternatives for their everyday meals (5–8). Other factors such as the low cost, convenience and taste have strengthened street food's popularity, which is currently consumed everyday by millions of people worldwide (6). In cities from central Asia, street food trade is a well-developed activity, being often organized in typical markets called *bazaars*. Street food vending sites are common, reflecting the high cultural and dietary importance of street food in the region (9–12).

Frequent consumption of foods outside the home has been reported as a risk factor for overweight and obesity (13, 14), as well as higher energy and fat intake and lower micronutrient intake (15). In line with other LMIC (16), in urban areas from central Asia, street food has been reported as source of foods frequently rich in total fat, saturated and *trans* fatty acids, as well as sodium. Availability data showed that although local foods remain common, globalized ultra-processed food products such as industrial snacks and soft drinks are frequently available (9–12, 17–19). However, to the best of our knowledge, no systematic characterization of the purchasing patterns of street food in these settings is available. A recent scoping review also showed that, even though research on street food has been growing, its purchase and consumption are amongst the least studied topics (20). As such, the objective of this study was to describe the patterns of street food purchase in the main urban areas of four central Asian countries, Tajikistan, Kyrgyzstan, Turkmenistan and Kazakhstan, considering the food items bought and their nutritional composition, overall and according to time of the day and place of purchase. In this study, we go beyond the simple characterization of street food purchases and their nutritional value, by identifying where and when specific patterns of purchase emerge. With this approach, we hope to contribute to a more in-depth understanding of the street food buying practices in these cities (both in terms of meal context and city location), thus allowing the design of public health strategies customized not only to the reality of these food environments, but also to their customers' food habits.

## Materials and Methods

### Overview

This study was implemented in the context of the FEEDCities project, which is based on a stepwise standardized characterization of the street food environment in cities from Central Asia and Eastern Europe. The general methodology of this research project is published elsewhere (21). For the purpose of this study, four cross-sectional evaluations of street food customers were conducted in the largest and most urbanized cities of Tajikistan, Kyrgyzstan, Turkmenistan and Kazakhstan (22), respectively *Dushanbe* (between April and May 2016), *Bishkek* (June and July 2016), *Ashgabat* (October 2016) and *Almaty* (August 2017).

### Eligibility Criteria

Street food was defined by the Food and Agriculture Organization (FAO) and the World Health Organization (WHO), as “ready-to-eat foods and beverages prepared and/or sold by vendors or hawkers especially in the streets and other similar places” (23, 24). Eligible street food vending sites were those which sold street food in accordance to this definition, including both fixed and mobile units selling directly to the streets. As such, food establishments within four permanent walls or permanent storefront business not selling directly to the street, vending sites selling exclusively non-prepared fresh fruits, vegetables or other raw foods not ready-to-eat, such as fish or meat, or food stalls and carts that are part of permanent stores or licensed establishments, were excluded from the study.

Any customer approaching one selected street food vending site to buy ready-to-eat foods and/or beverages during a pre-specified time period was eligible for the study.

### Sampling Procedure

In the four cities assessed, all public markets were identified (*n* = 89), in accordance to information provided by local authorities and collected during preliminary field visits, as described elsewhere (9–12, 17, 18). Ten markets were randomly selected in each city, except for *Ashgabat*, where four markets were selected by local authorities. To identify the study area in each city, buffers with a diameter of 500 m were drawn around the centroid of each selected market, that way covering the whole market and surrounding areas. Within each study area, all publicly accessible streets were canvassed by pairs of field researchers, who identified all street food vending sites eligible for the study.

Taking into account the density of vending sites encountered in each study area and the expected overall number of customers, a systematic selection of the vending sites was performed, in which one out of every ten (*Dushanbe*), one out of every four (*Bishkek* and *Almaty*) and all vending sites (*Ashgabat*) were selected for customer observation.

Each selected vending site was observed for a maximum period of 15 min to collect data on the street food items purchased. Each observation ended when four consecutive customers were observed, or whenever the maximum period was reached. When no customers were observed within the pre-specified period, field researchers moved on to the next selected vending site. Observations were performed on consecutive days of the week, and covering all businesses' working hours, from 08:00 to 17:00.

### Data Collection

Data was collected by direct observation of each selected street food vending site. Observations were performed by local field researchers, located at enough distance not to disturb the vendors' regular activity or the customers' usual behavior.

Data was collected by two observers who, for each customer observed, independently registered the foods and/or beverages purchased and respective quantities. Prior to data collection, all field researchers were trained (including both theoretical, practical and field training) aiming at the improvement and standardization of observations.

Inter-observer concordance was evaluated using Cohen's kappa (*K*) with 95% confidence interval (95% CI), being high to very high, regarding the foods and/or beverages purchased (97.6% agreement, *K* = 0.98, 95% CI 0.97–0.99) and their quantities (95.3% agreement, *K* = 0.94, 95% CI 0.92–0.97), as detailed elsewhere (25). Disagreements on the foods and/or beverages purchased or their quantities were eliminated using a set of standardized criteria: (1) if the two observers registered two different foods and/or beverages, the conflicting items were checked for their availability on the corresponding vending site (if only one of the conflicting items was available, this observation was assumed; if the conflicting items were both available or not available, both observations were disregarded); (2) if the two observers registered the same food and/or beverage, but with different degrees of specificity, the broadest observation was assumed; (3) if the two observers registered a different number of foods and/or beverages for the same customer, the observation with the higher number of food items was assumed, unless the conflicting item(s) was(were) not available in the corresponding vending site; and (4) if the two observers registered the same food and/or beverage, but different quantities, the average quantity between the two observations was assumed.

Based on the WHO nutrient profile model (26) and considering similarities of nature and/or composition, the purchased foods and beverages were grouped into six sub-categories each: (1) savory pastries and snacks; (2) main dishes; (3) sweet pastries and confectionery; (4) bread; (5) sandwiches; (6) fruit; (7) tea and coffee; (8) soft drinks and juices; (9) traditional beverages (non-alcoholic); (10) alcoholic beverages; (11) milk; and (12) water. Foods and beverages were also classified as *homemade* (foods and beverages that were prepared and/or cooked at home or in the street, even if using industrial ingredients) or *industrial* (foods and beverages that were produced by the food industry and sold as such, with no further preparation).

For each vending site observed, Global Positioning System (GPS) coordinates were recorded, as well as time of beginning and end of observation. The selected markets were classified as central or peripheral, according to their distance (below or above 3 km, respectively) to a city center reference point, assigned in each setting taking into account information provided by the respective WHO Country Offices and local collaborating institutions. The duration of the observation was calculated for each vending site, as well as the customers' influx (which was computed as the number of customers observed per 10 min of observation) and the food items buying rate (number of foods and/or beverages purchased per 10 min of observation). Purchases were then categorized taking into account time of the day, on an hourly basis (i.e. [09:00–10:00], [10:00–11:00], etc.) and city location (city center vs. periphery).

### Nutritional Composition Estimation

The nutritional composition of all purchased street foods and beverages was estimated, either using data from chemical analysis or using information provided by food labels, standardized recipes and food composition tables.

During the first step of the research project, which assessed street food availability, the most frequently available street foods and beverages in each city were documented (9–12, 17, 18). Taking this information into account, chemical analyses of the most common street foods were performed, as previously described in detail (9–12, 17, 18), and included macronutrients (protein, total fat and carbohydrates by difference), fatty acids (saturated, monounsaturated, polyunsaturated and *trans*), sodium and potassium, as well as energy calculated using the Atwater general factors, following validated and standardized methodology (27). In *Ashgabat*, exceptionally, the samples collected were not analyzed for protein and carbohydrates contents, those being estimated using food composition tables, standardized recipes or food labels, as described below.

For the foods and beverages which were not sampled and subjected to chemical analysis, nutritional composition was estimated using: (1) food labels of the brands most commonly available in each setting, in the case of industrial foods or beverages (e.g. chocolate, soft drinks); (2) standardized recipes from each city, in the case of prepared and/or cooked homemade foods or beverages; (3) food composition tables, namely the Food Composition Table for Pakistan (28) and the Turkish National Food Composition Database (29), in the case of *in natura* foods or beverages (e.g. fruits, nuts) or recipe ingredients. Energy, protein, carbohydrates and total fat were estimated for all these foods and beverages, as well as sodium for industrial and *in natura* foods. Sodium contents of homemade foods and beverages were not estimated, due to the great variation in the quantities of salt added during their preparation or cooking.

Among all the foods and beverages purchased (*n* = 852), the nutritional composition of 582 (68.3%) was estimated using data from chemical analysis, and the remaining (*n* = 270, 31.7%) using food labels, standardized recipes and food composition tables. The nutritional value of the street food purchases of each customer was then computed by summing up the estimated energy, macro- and micronutrients of all foods and beverages purchased by the same customer on a single occasion.

### Statistical Analysis

Absolute and relative frequencies were used to describe the foods and beverages purchased. Customers' influx, food items buying rate and number of food items purchased per customer, as well as the nutritional composition of the purchases were described through median and percentiles 25 and 75. Pearson's Chi-squared test was used to compare the street food purchases throughout the day and by city location. Mann-Whitney's and Kruskal–Wallis tests were used to compare the customers' influx, food items buying rate and number of food items purchased per customer, as well as the nutritional composition of the purchases, either throughout the day or by city location, respectively. Differences were considered statistically significant when the critical level of significance (*p*) was less than 0.05. Statistical analysis was performed using *Stata*® version 15.0 (StataCorp., College Station, TX, USA).

## Results

A total of 390 street food vending sites were observed (71 in Tajikistan, 137 in Kyrgyzstan, 93 in Turkmenistan and 89 in Kazakhstan), corresponding to the completion of 4,769 min of observation. A total of 714 customers were observed, who bought 852 foods and beverages. In each vending site, a median of 2 customers were observed over a median period of 15 min.

**Figure 1:**
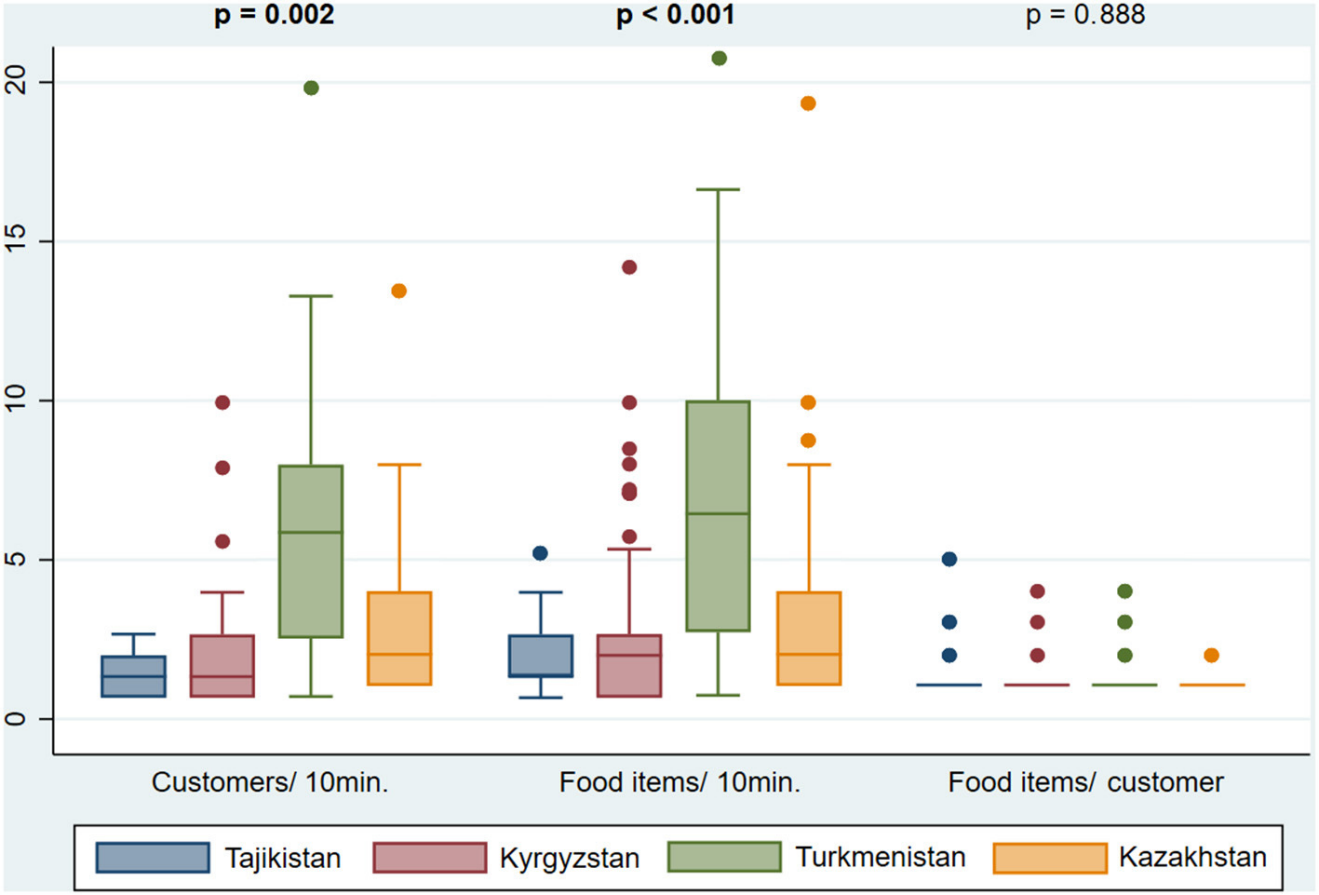
Customers' influx (customers/10 min), food items buying rate (food items/10 min) and number of food items purchased per customer (food items/ customer), in street food vending sites observed in *Dushanbe* (Tajikistan), *Bishkek* (Kyrgyzstan), *Ashgabat* (Turkmenistan) and *Almaty* (Kazakhstan). Values in bold represent statistically significant differences according to Kruskal–Wallis test with a significance level of 0.05.

**Figure 2:**
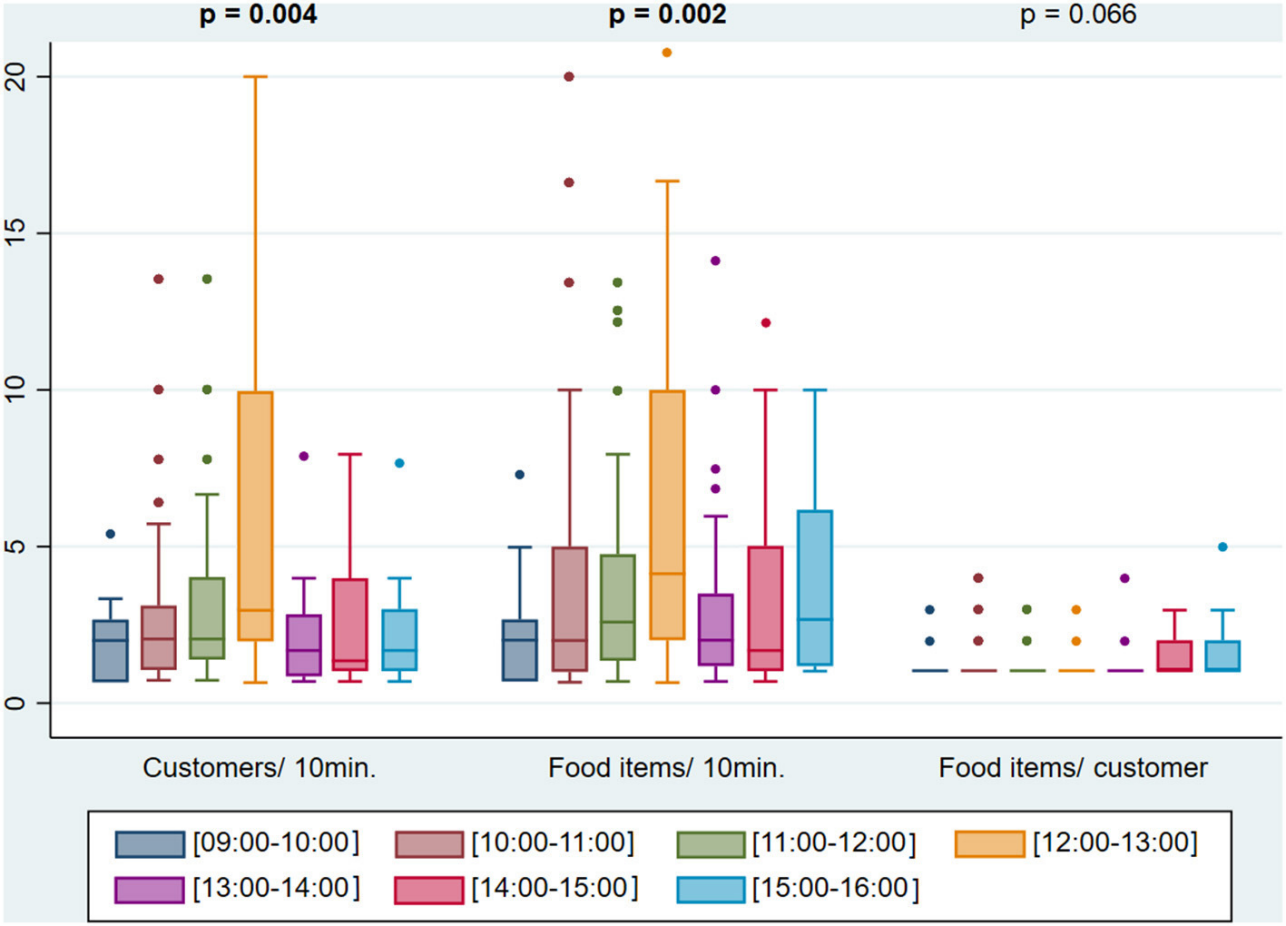
Customers' influx (customers/10 min), food items buying rate (food items/10 min) and number of food items purchased per customer (food items/ customer) throughout the day. Values in bold represent statistically significant differences according to Kruskal–Wallis test with a significance level of 0.05.

**Figure 3:**
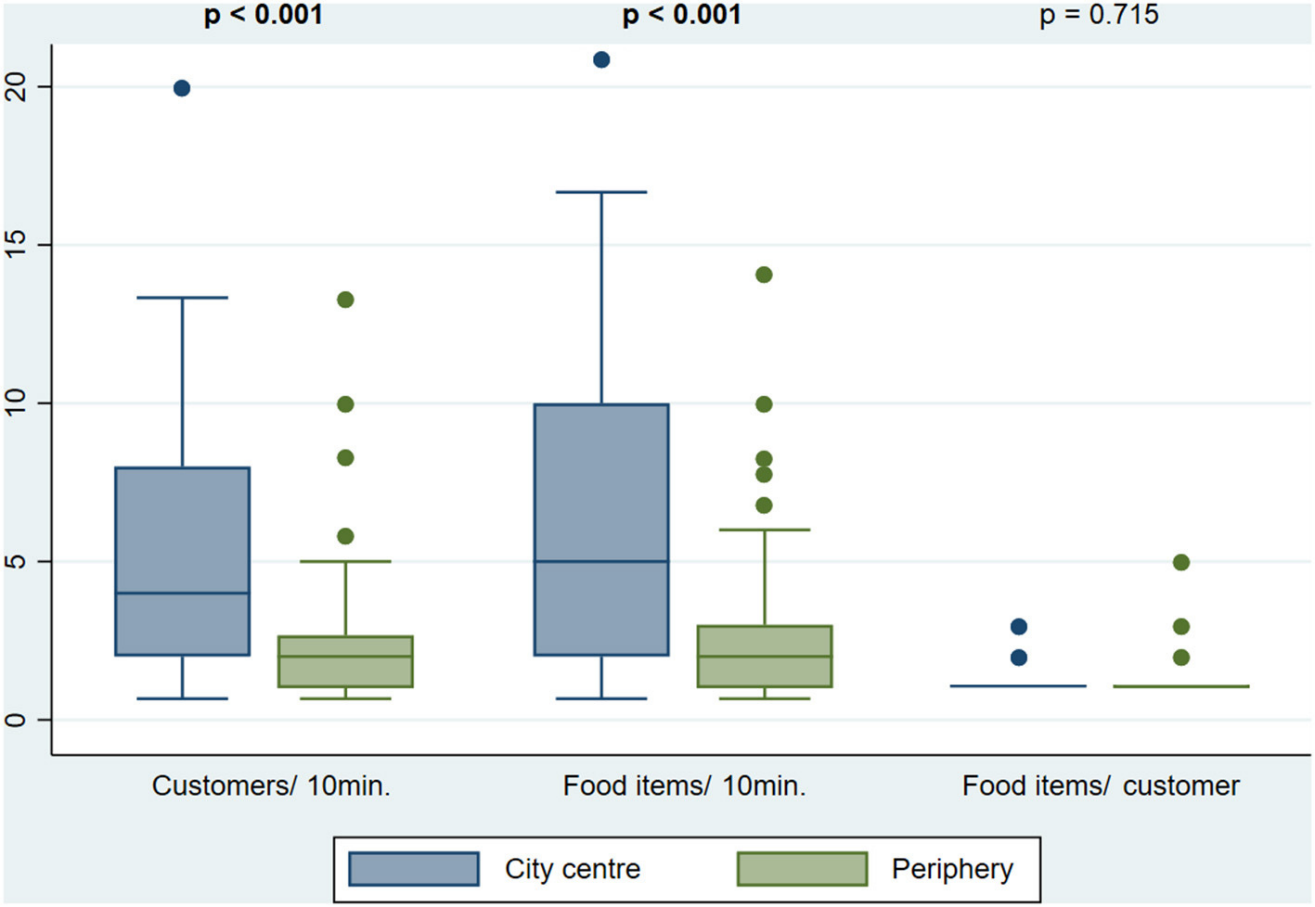
Customers' influx (customers/10 min), food items buying rate (food items/10 min) and number of food items purchased per customer (food items/ customer) by city location. Values in bold represent statistically significant differences according to Mann-Whitney's test with a significance level of 0.05.

The median customers' influx and food items buying rate varied across countries, being the lowest in Tajikistan (1.3 customers and 1.3 food items per 10 min of observation, respectively) and the highest in Turkmenistan (5.9 customers and 6.4 food items per 10 min of observation, respectively). The number of food items purchased by customer was similar in all countries, with a median of 1.0 (Figure 1). No customers were observed from 08:00 to 09:00 and from 16:00 to 17:00. Throughout the day, customers' influx and food items buying rate varied, being highest between 12:00 and 13:00 (median: 3.0 customers/10 min; 4.1 food items/10 min). The number of food items purchased by customer was constant (median of 1 in all hours of the day), although between 15:00 and 16:00, a higher proportion of customers (47.1%) purchased two or more foods and/or beverages (Figure 2). City centers showed higher customers' influx (4.0 vs. 2.0 customers/10 min, *p* < 0.001) and food items buying rate (5.0 vs 2.0 food items/10 min, *p* < 0.001) than the peripheries (Figure 3).

**Figure 4:**
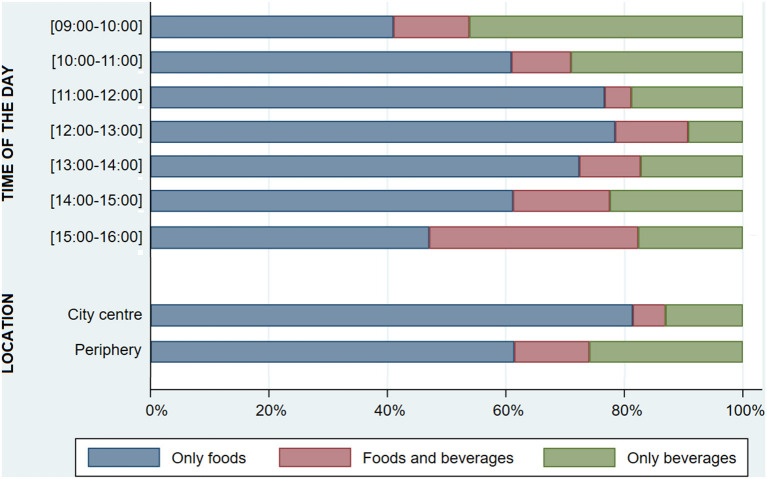
Proportion of customers purchasing foods and/or beverages, throughout the day and by city location (*n* = 714).

**Figure 5:**
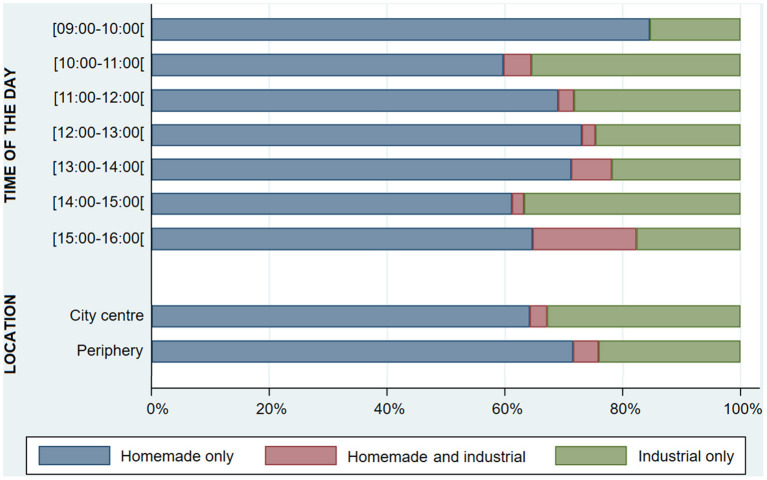
Proportion of customers purchasing homemade and/or industrial food items, throughout the day and by city location (*n* = 714).

Purchase of foods was the highest between 12:00 and 13:00 (90.8%), whereas beverages were more frequently purchased in both the beginning (59.0%) and end of the day (52.9%). Between 15:00 and 16:00, it was observed a greater frequency of joint purchase of foods and beverages (35.3%). The purchase of foods was higher in the city center (86.8 vs. 74.2%, *p* = 0.004) while beverages were more frequently bought in the periphery (38.6 vs. 18.7%, *p* < 0.001; Figure 4). Purchase of homemade food items surpassed 80% from 9:00 to 10:00 and 15:00 to 16:00, while industrial products buying frequency was the highest between 10:00 and 11:00 (40.2%), and between 14:00 and 15:00 (38.8%). Customers purchasing industrial food items were significantly more frequent in the city center (36.2 vs. 28.7%, *p* = 0.004) (Figure 5). From all customers buying foods and beverages together (*n* = 71), more than half (59.2%) purchased both foods and beverages homemade (mostly savory pastries with coffee or tea) and one out of every three (35.2%) bought one or more homemade foods with one industrial beverage (mostly main dishes, sandwiches or savory pastries, plus soft drinks).

The distribution of groups of foods and beverages throughout the day and by city location is presented in Table 1. Among customers who bought foods, savory pastries and snacks were most frequently purchased from 09:00 to 11:00 and 15:00 to 16:00, main dishes between 11:00 and 13:00, sweets pastries and confectionery from 13:00 to 15:00, bread from 09:00 to 10:00 and 12:00 to 13:00, and sandwiches between 15:00 and 16:00. Among the purchases which included beverages, tea and coffee were most frequently purchased in the first time period, soft drinks and juices between 15:00 and 16:00, and water from 12:00 to 14:00. Bread and sweet pastries and confectionery were more commonly bought in the city center, while the purchases of savory pastries and snacks, as well as main dishes, were higher in the periphery.

**Table 1:** Distribution of the street food purchases throughout the day and by city location, by food and beverage groups.

	***N*** **^a^**	**Savory pastries and snacks**	**Main dishes**	**Sweet pastries and confectionery**	**Bread**	**Sandwiches**	**Fruit**	***N*** **^b^**	**Tea and coffee**	**Soft drinks and juices**	**Non-alcoholic traditional beverages^**c**^**	**Water**	**Alcoholic beverages^**d**^**	**Milk**
		***n*** **(%)**							***n*** **(%)**					
**Time of the day**														
**[09:00–10:00]**	21	11 (52.4)	2 (9.5)	1 (4.8)	6 (28.6)	2 (9.5)	0 (0.0)	23	16 (69.6)	4 (17.4)	3 (13.0)	0 (0.0)	0 (0.0)	0 (0.0)
**[10:00–11:00]**	120	54 (45.0)	23 (19.2)	25 (20.8)	13 (10.8)	10 (8.3)	2 (1.7)	66	25 (37.9)	24 (36.4)	5 (7.6)	11 (16.7)	2 (3.0)	1 (1.5)
**[11:00–12:00]**	181	48 (26.5)	52 (28.7)	47 (26.0)	20 (11.0)	18 (9.9)	3 (1.7)	52	9 (17.3)	20 (38.5)	14 (26.9)	3 (5.8)	6 (11.5)	0 (0.0)
**[12:00–13:00]**	118	23 (19.5)	35 (29.7)	20 (16.9)	39 (33.1)	4 (3.4)	0 (0.0)	28	14 (50.0)	4 (14.3)	4 (14.3)	6 (21.4)	0 (0.0)	0 (0.0)
**[13:00–14:00]**	72	15 (20.8)	12 (16.7)	22 (30.6)	15 (20.8)	18 (25.0)	2 (2.8)	24	8 (33.3)	3 (12.5)	5 (20.8)	5 (20.8)	3 (12.5)	0 (0.0)
**[14:00–15:00]**	38	8 (21.1)	9 (23.7)	13 (34.2)	9 (23.7)	1 (2.6)	1 (2.6)	19	9 (47.4)	7 (36.8)	2 (10.5)	0 (0.0)	0 (0.0)	2 (10.5)
**[15:00–16:00]**	14	7 (50.0)	3 (21.4)	0 (0.0)	0 (0.0)	4 (28.6)	0 (0.0)	9	0 (0.0)	8 (88.9)	0 (0.0)	0 (0.0)	1 (11.1)	0 (0.0)
*p* **-Value**		**<0.001**	**0.007**	**0.010**	**<0.001**	**0.027**	0.720		**<0.001**	**<0.001**	0.080	**0.020**	0.076	**0.032**
**City location**														
**City center**	237	47 (19.8)	42 (17.7)	76 (32.1)	58 (24.5)	21 (8.9)	5 (2.1)	51	17 (33.3)	18 (35.3)	7 (13.7)	8 (15.7)	1 (2.0)	2 (3.9)
**Periphery**	327	119 (36.4)	94 (28.8)	52 (15.9)	44 (13.5)	26 (8.0)	3 (0.9)	170	64 (37.6)	52 (30.6)	26 (15.3)	17 (10.0)	11 (6.5)	1 (0.6)
*p* **-Value**		**<0.001**	**0.025**	**0.001**	**0.025**	0.854	0.809		0.614	0.611	0.865	0.538	0.625	0.718

*Values in bold represent statistically significant differences according to Pearson's Chi-squared test with a significance level of 0.05*.

*The proportions presented are relative to the number of customers purchasing foods (in the case of food groups) or beverages (in the case of beverage groups) in each time period or city location*.

^a^*Number of customers purchasing foods (n = 564)*.

^b^*Number of customers purchasing beverages (n = 221)*.

^c^*Non-alcoholic traditional beverages included ayran (dairy-based fermented beverage made from sheep's milk), chalap (beverage made from fermented milk, salt and carbonated water;), dugob (fermented beverage made with sour milk or buttermilk), kefir (fermented milk drink prepared by inoculating cow, goat or sheep milk with kefir grains), maksym (fermented beverage made from grain, usually malt), tamshan (mix of maksym and chalap), kozhe (cold drink made by boiling rice, millet or pearl barley with a mixture of dairy products such as ayran or kefir) and yogurt*.

^d^*Alcoholic beverages included beer, vodka and some traditional beverages with a low alcohol content, such as kvass (a fermented beverage made from rye bread), bozo (a fermented beverage made from millet) and kymyz (a fermented product made from mare's milk)*.

The observed customers purchased a median amount of 120 g of foods and 200 g of beverages. Table 2 presents the estimated nutritional composition of the purchases observed, including foods and beverages, throughout the day and by city location. Between 11:00 and 12:00, purchases were more energy-dense and richer in protein, carbohydrates and fat, the opposite being observed from 09:00 to 10:00. Saturated and *trans* fatty acids contents were both highest between 13:00 and 14:00. Purchases made in the city center were significantly more energy-dense and richer in all macronutrients, while in the periphery purchases showed higher saturated and *trans* fatty acids contents. The temporal and spatial distribution of the estimated nutritional composition of the street food purchases observed, without beverages, is presented in Supplementary Table 1.

**Table 2:** Estimated nutritional composition of the street food purchases observed (foods and beverages combined), throughout the day and by city location (*n* = 714).

	**Amount purchased**	**Energy density**	**Protein**	**Carbohydrates**	**Total fat**	**SFA**	**MUFA**	**PUFA**	**TFA**	**Sodium**	**Potassium**
	**g**	**kcal/100 g**	**g/100 g**	**g/100 g**	**g/100 g**	**g/100 g of fat**	**g/100 g of fat**	**g/100 g of fat**	**g/100 g of fat**	**mg/ 100 g**	**mg/ 100 g**
**Time of the day**											
**[09:00–10:00]**	200 (135–200)	88 (16–314)	2.5 (0.0–11.7)	15.6 (4.0–33.1)	1.0 (0.0–9.5)	34.1 (20.7–42.9)	27.1 (25.5–32.2)	35.2 (19.4–49.7)	1.98 (1.65–5.46)	500 (329–599)	109 (51–144)
**[10:00–11:00]**	200 (100–350)	228 (48–557)	6.3 (0.8–17.5)	26.0 (10.0–79.0)	6.2 (0.3–18.7)	36.0 (19.4–45.3)	28.8 (26.2–33.6)	29.4 (18.5–49.6)	1.94 (1.19–2.59)	597 (356–1,136)	309 (151–529)
**[11:00–12:00]**	200 (96–265)	310 (126–585)	9.1 (4.3–17.0)	45.1 (13.5–70.5)	10.0 (2.2–20.1)	35.5 (20.7–46.5)	29.3 (26.2–33.6)	34.5 (18.5–49.3)	1.67 (0.95–3.65)	490 (348–1,136)	279 (145–454)
**[12:00–13:00]**	120 (100–286)	286 (128–404)	8.5 (3.9–11.0)	40.6 (16.7–56.8)	4.5 (1.7–15.3)	22.3 (15.8–41.0)	26.6 (18.0–32.1)	49.4 (19.4–66.3)	1.08 (0.67–2.68)	467 (394–917)	170 (150–443)
**[13:00–14:00]**	150 (120–278)	233 (130–329)	9.0 (3.9–12.5)	33.3 (12.9–57.6)	2.5 (1.5–13.2)	38.6 (19.4–60.3)	27.3 (23.9–29.0)	24.9 (7.8–51.9)	3.33 (0.67–5.36)	472 (141–620)	166 (136–303)
**[14:00–15:00]**	156 (96–300)	274 (89–522)	5.6 (2.5–10.6)	42.1 (12.0–65.1)	7.7 (0.3–20.9)	31.1 (21.8–39.6)	28.3 (24.1-−33.0)	32.0 (18.0–52.3)	2.50 (1.08–7.57)	497 (402–1,041)	244 (121–446)
**[15:00–16:00]**	265 (172-−494)	233 (117–286)	4.3 (2.7–8.5)	27.9 (17.3–40.9)	10.1 (4.0–13.4)	32.1 (23.1–37.5)	27.7 (24.7–31.8)	37.5 (28.6–51.1)	1.39 (1.08–2.14)	484 (197–1,264)	227 (122–461)
*p* **-Value**	0.458	**0.001**	**0.011**	**0.008**	**<0.001**	**0.008**	**<0.001**	**0.002**	**<0.001**	0.256	**<0.001**
**City location**											
**City center**	120 (96–265)	308 (206–552)	9.1 (4.0–17.1)	56.8 (20.6–80.2)	10.0 (1.7–20.1)	27.6 (19.4–41.6)	27.8 (24.0–32.0)	37.7 (18.5–52.9)	1.20 (0.81–2.15)	484 (435–1,042)	305 (150–507)
**Periphery**	200 (117–306)	216 (77–419)	6.6 (2.4–12.3)	26.0 (10.0–57.6)	4.6 (0.6–14.1)	33.9 (20.4–42.9)	27.9 (25.5–32.9)	34.5 (18.5-−51.1)	2.25 (1.29–5.46)	490 (294–980)	205 (125–402)
*p* **-Value**	**<0.001**	**<0.001**	**0.003**	**<0.001**	**<0.001**	**0.005**	0.055	0.094	**<0.001**	0.254	**<0.001**

*SFA, saturated fatty acids; MUFA, monounsaturated fatty acids; PUFA, polyunsaturated fatty acids; TFA, trans fatty acids*.

*All values are presented as median (P25–75). Values in bold represent statistically significant differences according to Kruskal–Wallis (throughout the day) and Mann-Whitney's tests (by city location) with a significance level of 0.05*.

## Discussion

Street food purchase in these four central Asian urban centers was frequent and reached its maximum around lunchtime. Purchases consisted of a diverse set of both local and westernized foods and beverages, and varied throughout the day and by city location. Tea, coffee, bread and savory pastries were frequently purchased in the early morning; bread, main dishes and savory pastries at lunch; and industrial snacks (both savory and sweet) and beverages (mainly soft drinks and juices) were mostly bought in the mid-morning and mid-afternoon periods. Purchase of industrial foods and beverages, sweet pastries and confectionery and bread was more frequent in the city center, while main dishes, savory pastries and snacks were mostly bought in the periphery. Energy and macronutrient density was highest around lunchtime and lowest in the early morning. Purchases were smaller but more energy-dense in city centers, and higher in SFA and TFA in the peripheries.

Customers' influx and food items buying rate was the highest between 12:00 and 13:00, which was also when the frequency of purchase of foods reached its peak. Nine out of 10 customers purchased foods during this time period, mostly bread, main dishes and savory pastries, suggesting that these food groups may be frequently consumed by street food customers at lunch. This is in line with evidence showing that street food is often consumed as a substitute for home-cooked main meals (6, 30), and may be closely associated with changes in time allocation patterns occurring in Asian LMIC, toward an increasingly accelerated and much more work-oriented daily life (31, 32). One important aspect of the urban lifestyle is the increase in the number of time-limited consumers, which in turn increases the demand for convenience foods (31). Besides convenience, street food also offers affordable and fulfilling food options, gaining momentum in LMIC over more formal alternatives such as restaurants (6, 8, 16, 30). Customers' influx and food items buying rate was also higher in the city centers when compared to their outskirts, which was expected. These urban centers, similarly to other LMIC, have undergone abrupt growth and development, with an accelerated construction of infrastructures and increased concentration of companies, services and people. As a consequence, a large increase in the size of the labor force has occurred, especially in these city centers (2, 33). As reported in other LMIC, street food customers are usually urban workers but rural or peri-urban dwellers, who often need to make long commutes between house and the workplace on a daily basis (34, 35), and therefore have little availability to travel back home for their meals during the day. Accordingly, street food sites are often located near people gathering spaces, such as working places or main public transportation stations (6, 36), where it is expected that there will be a greater influx of people and higher demand for these ready-to-eat food options.

Frequency of purchase of industrial food products was highest in the periods between 10:00 and 11:00, and between 14:00 and 15:00. These acquisitions were mostly constituted of savory snacks, sweet pastries and confectionery, as well as soft drinks and juices. This suggests that these industrial foods and beverages may be commonly preferred by the street food customers as mid-morning or mid-afternoon snacks. Sweet pastries and confectionery were most frequently purchased between 13:00 and 15:00 which may as well correspond to an after lunch time period, suggesting that some of these foods may have also been consumed in the context of a dessert. However, we cannot exclude the possibility of sweet pastries being consumed as the main meal, since these foods are fulfilling and have a low cost per calorie (37), and in contexts of economic restriction, they may be seen by consumers as a good option to supply energy needs. Furthermore, food-based dietary guidelines and nutrition education programmes are still lacking in these countries, so there may be a lack of knowledge about which foods are suitable to consume at different meals throughout the day. Soft drinks and juices were less purchased between 12:00 and 14:00, and during this time period mainly tea and water were bought. This is consistent with the types of beverages that are traditionally consumed during lunchtime (38), and may indicate a lower preference of street food consumers for soft drinks during this meal.

Frequency of purchase of industrial foods was also higher in the city centers, and was mostly comprised of sweet pastries and confectionery (mainly candies, ice-cream and chocolates). This can either be explained by spatial variations on street food buying habits, or be a reflection of these foods' availability on central vs. peripheral vending sites. According to information from the first step of the FEEDcities project, there was a similar proportion of vending sites selling sweet pastries and confectionery in central and peripheral markets (unpublished data), which indicates that in city centers there seems to be a greater preference of street food consumers for these westernized foods. Industrial beverages, such as soft drinks and juices, were equally and frequently bought in both city subareas, suggesting that their consumption might be an already widely implemented habit. The acquisition of those ultra-processed sweetened beverages occurred at all hours of the day, almost always surpassing that of other common drinks, such as water, milk and non-alcoholic traditional beverages, the latter mostly comprised of fermented milk-based drinks (e.g. *kefir, ayran, chalap*). These results make us hypothesize that soft drinks and industrialized juices may be consumed as a substitute for others with a potentially healthier profile. Our results are in line with the increasing availability and consumption of ultra-processed foods and beverages that is happening in the region, arising from quick economic and technological development, as well as globalization, which have been transforming food systems in Asian LMIC (39, 40). The consumption of ultra-processed foods and beverages have been increasingly recognized as a risk factor for a set of adverse health effects, including overweight, obesity, cancer, type II diabetes, cardiovascular diseases and all-cause mortality (41, 42), being also associated to lower dietary quality by providing excessive amounts of fat, sugar, and/or sodium in an energy-dense and nutrient-poor food matrix (43). This is particularly concerning since excess weight, as well as mortality and morbidity from non-communicable diseases, have been increasing during the last decades in central Asia. According to data from the Global Nutrition Report 2016, in this region overweight affected almost half of the adult population (49.8% of women and 49.2% of men) (44). In 2019, non-communicable diseases were responsible for approximately 84.7% of total deaths and 72.9% of total disability-adjusted life years in the region (45).

The highest purchase of homemade foods and beverages was observed between 09:00 and 10:00, indicating a preference by street food customers for home-like food options in the context of a breakfast. Nearly half of customers purchasing foods bought savory pastries during this time period, although they are usually consumed at any time of the day (38), which is consistent with our results. Bread, tea and coffee are also purchased at almost all hours of the day, but mainly between 09:00 and 10:00, and 12:00 and 13:00. These food items are traditionally consumed in the context of breakfast, or as part of lunch (38), our results indicate that this habit seems to remain amongst the street food consumers of these cities. Purchase of sandwiches was less common, occurring mostly in the afternoon (in the periods from 13:00 to 14:00 and from 15:00 to 16:00). Although their consumption in the region is not unusual (38), sandwiches can be potentially easier to prepare at home, and transported without losing quality, which may explain in part the lower purchase. On the other hand, the time periods in which there was a higher purchase of sandwiches suggest that this type of foods may be consumed by street food customers mainly at lunch as a main course [as reported elsewhere (17)] or as an afternoon snack. The sandwiches bought were most frequently filled with cheese, sausage or meat [e.g. *shawarma* (kebab), hamburger, hot-dog]. Similarly, savory pastries and main dishes were very often meat-based: the most frequently purchased savory pastries were *sambusa, piroshky* and *chebureki*, which are all traditional pastries, usually fried or baked and filled with meat; whereas the most commonly bought main dishes were *plov* (rice cooked in a seasoned broth with a mixture of spices, usually with meat and vegetables) and *shashlik* (skewered grilled cubes of meat, alone or with alternating pieces of meat, fat and vegetables). This reflects the importance of meat in this region's gastronomy, as it can be present in any meal and it is the primary source of protein in central Asian diets (38).

Fruit was the least frequently bought street food in all cities studied. This may be a direct reflection of the low ready-to-eat fruit availability that was found in the selected street food vending sites, which ranged from 1.0% in *Almaty* to 4.5% in *Dushanbe* (9–12, 17, 18). The Global Burden of Disease Study (46) and the EAT-Lancet Commission (47) provided targets for fruit consumption, at 200 and 250 g of fruit per day, respectively. In Central Asia, data from the FAOSTAT food balances show that fruit supply has been consistently rising throughout the years, and in 2018 it reached 72.61 kg/capita/year (48), which corresponds to approximately 199 g/capita/day. Since fruit supply do not correspond directly to actual consumption, due to factors such as edible portions and food waste losses, these values indicate that overall consumption of fruit may still be inadequate. However, the growth in fruit availability in the region also suggests that it may be possible to reach these consumption targets in the near future. A study conducted in former Soviet Union countries (including Kyrgyzstan and Kazakhstan) found a positive correlation between the number of shops selling fruits and vegetables and their consumption (49). Improving access to fruits and vegetables by increasing their availability in the streets have also showed to have positive results in the consumption of these foods (49, 50). As such, there seems to be a window of opportunity to raise fruit consumption in these street food contexts through availability policies.

Regarding the nutritional composition of the purchases, the highest energy and macronutrient density was observed between 11:00 and 12:00, mostly due to a high level of purchase of main dishes and energy-dense sweets and savory pastries and snacks. On the contrary, purchases were poorer in energy and macronutrients from 09:00 to 10:00, which was also when the highest proportion of customers bought beverages, most of them with a very low nutritional value. Saturated fatty acids were the highest contributors to total fat in almost all hours of the day, reaching its highest content in purchases made between 13:00 and 14:00. *Trans*-fat was also high, and above the limit of 2 g/100 g of total fat (51, 52) between 13:00 and 15:00. These results can be explained by a high acquisition of sweets pastries and confectionery during these time periods. Some of these foods, particularly wafers, chocolate and ice-cream, were identified as rich sources of both saturated and *trans*-fat in these street food environments (9–12, 17, 18), which was particularly concerning due to their proximity to schools, as observed *in loco*. In the city centers, purchases were smaller but more energy-dense; however, in the periphery purchases had higher proportions of saturated and *trans*-fat, mostly due to the high percentage of customers buying savory pastries and snacks, as well as main dishes, some of them also providing high contents of those fatty acids (9–12, 17, 18).

Limitations of the present study should be discussed. We were unable to perceive purchasing variations throughout the week, or between week days and weekends. Although observation efforts comprised all days of the week (from Monday to Sunday), most street food markets were assessed during one or two consecutive days, due to logistical reasons. Future work could eliminate this limitation by assessing each market during a whole week. The fact that the observations were made during all days of the week was, nevertheless, an asset for this work, because it allowed us to ensure that variability over the 7 days of the week was covered when analyzing the patterns of purchase either throughout the day or by city location. Another limitation was that time of purchase may not directly mean time of consumption. However, all foods and beverages were ready-to-eat, purchased in small quantities and mostly unpackaged, suggesting that these purchases were made mostly for immediate consumption. Purchases including foods or beverages not ready-to-eat were not included in this study, thus eliminating purchases intended for household consumption.

To our knowledge, this is the first study describing street food purchasing patterns in cities from this region. The present results provide an overview of the street food buying habits in these cities, which may indirectly reflect consumption habits. Among the strengths of this study, the data collection through direct observation of customers by trained researchers instead of face to face interviews should be underlined, since it allowed us to minimize behavior alterations and social approval bias (53). We also highlight the high level of concordance between observers regarding the street food purchases. This reflects an underlying work of rigorous training, standardization of procedures and constant supervision, and resulted in a high reliability of these observational data. Finally, although generalizability of our results is very limited due to local gastronomic specificities, the FEEDCities standardized methodology (21) allows for data comparability among different cities, countries or regions.

## Conclusions

In conclusion, street food purchases in *Dushanbe* (Tajikistan), *Bishkek* (Kyrgyzstan), *Ashgabat* (Turkmenistan) and *Almaty* (Kazakhstan) varied widely across time and space, showing higher rates in city centers and during lunch. Different types of foods and beverages were bought at different frequencies throughout the day and by city location, reflecting specific patterns of purchase. The present study is the first to report on spatial and temporal distributions of street food purchases, that way contributing to a better understanding of the street food buying habits in these settings, which should be considered when designing public health strategies and defining targets of action more specific to the reality of these food environments, their customers and their gastronomic identity. Further research on the prevalence, frequency and determinants of street food consumption by these populations, would also be an important step in future work.

## Data Availability Statement

The raw data supporting the conclusions of this article will be made available by the authors upon request, without undue reservation.

## Ethics Statement

The studies involving human participants were reviewed and approved by Ethics Committee of the Institute of Public Health of the University of Porto. Written informed consent for participation was not required for this study in accordance with the national legislation and the institutional requirements.

## Author Contributions

MG, PM, JB, NL, and PP designed the study. GA, IL, and MG supervised the study implementation and data collection. OP and SC were responsible for the laboratorial analyses of the food samples collected in Tajikistan, Kyrgyzstan and Kazakhstan. CM coordinated the laboratorial analyses in Turkmenistan. SS, NL, and PP performed the analysis and interpretation of the results. SS drafted the first version of the manuscript. All authors critically revised the manuscript and gave their final approval of the manuscript.

## Funding

This work was supported by the World Health Organization Europe (WHO registration 2015/591370-0 and 2017/698514) and by the Ministry of Health of the Russian Federation. The EPIUnit – Instituto de Saúde Pública, Universidade do Porto (Ref. UIDB/04750/2020), the Investigation Unit LAQV/REQUIMTE (UID/QUI/50006/2020) and the ITR - Laboratório para a Investigação Integrativa e Translacional em Saúde Populacional (LA/P/0064/2020) are funded by Portuguese funds from FCT (Fundação para a Ciência e Tecnologia - Ministério da Ciência, Tecnologia e Ensino Superior). Individual grants attributed to SS (SFRH/BD/130650/2017) and GA (SFRH/BD/118630/2016) are funded by FCT and the Human Capital Operational Programme of the European Social Fund (POCH/FSE).

## Conflict of Interest

The authors declare that the research was conducted in the absence of any commercial or financial relationships that could be construed as a potential conflict of interest.

## Publisher's Note

All claims expressed in this article are solely those of the authors and do not necessarily represent those of their affiliated organizations, or those of the publisher, the editors and the reviewers. Any product that may be evaluated in this article, or claim that may be made by its manufacturer, is not guaranteed or endorsed by the publisher.
