# Corrigendum: Patterns of street food purchase in cities from Central Asia

**DOI:** 10.3389/fnut.2022.1005673

**Published:** 2022-08-19

**Authors:** Sofia Sousa, Inês Lança de Morais, Gabriela Albuquerque, Marcello Gelormini, Susana Casal, Olívia Pinho, Carla Motta, Albertino Damasceno, Pedro Moreira, João Breda, Nuno Lunet, Patrícia Padrão

**Affiliations:** ^1^EPIUnit - Instituto de Saúde Pública, Universidade do Porto, Porto, Portugal; ^2^Laboratório para a Investigação Integrativa e Translacional em Saúde Populacional (ITR), Porto, Portugal; ^3^Faculdade de Ciências da Nutrição e Alimentação da Universidade do Porto, Porto, Portugal; ^4^Nutrition, Physical Activity and Obesity Programme, Division of Noncommunicable Diseases and Life-Course, World Health Organization (WHO) Regional Office for Europe, Copenhagen, Denmark; ^5^LAQV/REQUIMTE, Laboratório de Bromatologia e Hidrologia, Faculdade de Farmácia, Universidade do Porto, Porto, Portugal; ^6^Departamento de Alimentação e Nutrição, Instituto Nacional de Saúde Doutor Ricardo Jorge (INSA), Lisboa, Portugal; ^7^Departamento de Ciências da Saúde Pública e Forenses e Educação Médica, Faculdade de Medicina da Universidade do Porto, Porto, Portugal; ^8^Faculdade de Medicina, Universidade Eduardo Mondlane, Maputo, Mozambique; ^9^Centro de Investigação em Atividade Física, Saúde e Lazer, Universidade do Porto, Porto, Portugal; ^10^WHO Regional Office for Europe, Athens, Greece

**Keywords:** street food, ready-to-eat food, purchasing patterns, food choice, nutritional value, Central Asia, low- and middle-income countries, nutrition transition

In the published article, there was an error in Figure 5 as published. The legend for the red bars was “Industrial only” and the legend for the green bars was “Homemade and industrial”. However, these legends were mistakenly switched. As such, the correct legend for the red bars is “Homemade and industrial” and the correct legend for the green bars is “Industrial only”. The corrected Figure 5 and its caption appear below.

**Figure 5:**
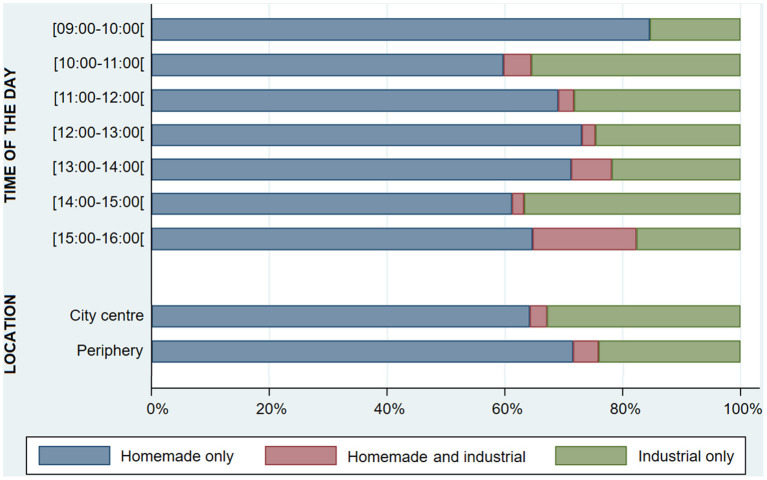
Proportion of customers purchasing homemade and/or industrial food items, throughout the day and by city location (*n* = 714).

The authors apologize for this error and state that this does not change the scientific conclusions of the article in any way. The original article has been updated.

## Publisher's note

All claims expressed in this article are solely those of the authors and do not necessarily represent those of their affiliated organizations, or those of the publisher, the editors and the reviewers. Any product that may be evaluated in this article, or claim that may be made by its manufacturer, is not guaranteed or endorsed by the publisher.

