# Supplementary material for: Patterns of Street Food Purchase in Cities From Central Asia
Source: Front Nutr. 2022 Jun 24;9:925771. doi: 10.3389/fnut.2022.925771 (PMC9263728; doi:10.3389/fnut.2022.925771)
Supplement: Supplementary file 1 [file Table_1.DOCX]

Supplementary material

|  | **Amount purchased** | **Energy density** | **Protein** | **Carbohydrates** | **Total fat** | **SFA** | **MUFA** | **PUFA** | **TFA** | **Sodium** | **Potassium** |
| --- | --- | --- | --- | --- | --- | --- | --- | --- | --- | --- | --- |
|  | g | kcal/100 g | g/100 g | g/100 g | g/100 g | g/100 g of fat | g/100 g of fat | g/100 g of fat | g/100 g of fat | mg/ 100 g | mg/ 100 g |
| **Time of the day** | |  |  |  |  |  |  |  |  |  |  |
| **[09:00-10:00[** | 120 (105-176) | 319 (275-520) | 11.7 (10.6-14.2) | 53.7 (31.1-68.6) | 13.6 (1.8-16.4) | 26.7 (20.7-42.9) | 27.0 (22.6-32.2) | 44.9 (19.4-57.0) | 2.21 (1.98-5.46) | 560 (490-959) | 144 (109-302) |
| **[10:00-11:00[** | 117 (52-265) | 420 (269-818) | 14.6 (8.0-23.8) | 58.6 (30.6-109.1) | 14.9 (7.2-30.1) | 33.6 (19.4-44.5) | 30.4 (26.2-33.2) | 28.3 (18.5-49.6) | 1.90 (1.19-2.40) | 892 (479-1787) | 329 (184-538) |
| **[11:00-12:00[** | 120 (94-265) | 449 (274-663) | 14.2 (7.0-18.7) | 57.6 (31.1-81.2) | 16.4 (6.2-26.3) | 32.9 (20.5-46.5) | 29.5 (26.2-33.6) | 35.7 (18.5-49.3) | 1.66 (0.90-2.79) | 611 (435-1216) | 301 (205-525) |
| **[12:00-13:00[** | 120 (96-186) | 286 (262-359) | 14.0 (6.4-16.5) | 56.8 (29.6-65.3) | 7.0 (1.7-24.1) | 22.3 (15.8-41.0) | 26.6 (18.0-32.1) | 49.4 (19.4-66.3) | 1.08 (0.67-2.68) | 467 (435-994) | 205 (150-461) |
| **[13:00-14:00[** | 126 (77-211) | 287 (207-479) | 11.0 (6.2-17.2) | 50.5 (29.6-61.8) | 5.9 (1.7-16.4) | 39.2 (19.4-60.3) | 27.3 (23.9-29.0) | 26.1 (7.8-51.9) | 3.10 (0.67-5.36) | 490 (169-887) | 167 (147-317) |
| **[14:00-15:00[** | 120 (92-176) | 328 (274-616) | 10.5 (6.4-14.5) | 57.2 (31.1-116.0) | 16.2 (6.3-26.0) | 28.9 (21.0-39.6) | 28.2 (23.9-32.2) | 33.0 (16.6-53.6) | 1.87 (1.08-7.65) | 505 (467-1052) | 303 (139-461) |
| **[15:00-16:00[** | 192 (100-265) | 290 (258-585) | 9.9 (8.5-14.9) | 35.5 (28.8-72.2) | 15.0 (11.5-24.1) | 32.1 (23.1-37.5) | 27.6 (24.7-31.8) | 37.5 (28.6-51.1) | 1.37 (1.08-2.14) | 777 (541-1533) | 353 (279-461) |
| **p** | 0.487 | **0.018** | **0.023** | 0.122 | **<0.001** | **0.019** | **<0.001** | **0.005** | **0.002** | **<0.001** | **<0.001** |
| **City location** |  |  |  |  |  |  |  |  |  |  |  |
| **City centre** | 117 (81-212) | 424 (276-663) | 11.0 (6.4-17.5) | 57.6 (39.9-108.9) | 14.0 (4.7-27.0) | 27.6 (19.1-41.6) | 27.8 (24.0-32.0) | 37.7 (18.9-52.9) | 1.20 (0.81-2.15) | 541 (467-1136) | 316 (151-536) |
| **Periphery** | 124 (96-265) | 319 (233-622) | 11.1 (7.2-17.6) | 45.1 (26.0-72.2) | 11.7 (3.8-24.1) | 31.2 (20.4-42.9) | 28.1 (25.7-32.6) | 37.3 (18.5-51.2) | 2.07 (1.19-5.36) | 560 (394-1136) | 243 (147-461) |
| **p** | **0.003** | **0.027** | 0.241 | **<0.001** | 0.262 | 0.220 | 0.052 | **0.015** | **<0.001** | 0.828 | **0.007** |

**Supplementary Table 1.** Estimated nutritional composition of the street food purchases observed (without beverages), throughout the day and by city location (n=564, number of customers purchasing foods).

SFA, saturated fatty acids; MUFA, monounsaturated fatty acids; PUFA, polyunsaturated fatty acids; TFA, *trans* fatty acids.

All values are presented as median (P25-75). Values in bold represent statistically significant differences according to Kruskal-Wallis (throughout the day) and Mann-Whitney’s tests (by city location) with a significance level of 0.05.
